# Hybrid Directed Hypergraph Learning and Forecasting of Skeleton-Based Human Poses

**DOI:** 10.34133/cbsystems.0093

**Published:** 2024-03-20

**Authors:** Qiongjie Cui, Zongyuan Ding, Fuhua Chen

**Affiliations:** ^1^ Nanjing University of Science and Technology, Nanjing, China.; ^2^ Changzhou University, Changzhou, China.; ^3^ Department of Physical Science & Mathematics, West Liberty University, West Liberty, WV, USA.

## Abstract

Forecasting 3-dimensional skeleton-based human poses from the historical sequence is a classic task, which shows enormous potential in robotics, computer vision, and graphics. Currently, the state-of-the-art methods resort to graph convolutional networks (GCNs) to access the relationships of human joint pairs to formulate this problem. However, human action involves complex interactions among multiple joints, which presents a higher-order correlation overstepping the pairwise (2-order) connection of GCNs. Moreover, joints are typically activated by the parent joint, rather than driving their parent joints, whereas in existing methods, this specific direction of information transmission is ignored. In this work, we propose a novel hybrid directed hypergraph convolution network (H-DHGCN) to model the high-order relationships of the human skeleton with directionality. Specifically, our H-DHGCN mainly involves 2 core components. One is the static directed hypergraph, which is pre-defined according to the human body structure, to effectively leverage the natural relations of human joints. The second is dynamic directed hypergraph (D-DHG). D-DHG is learnable and can be constructed adaptively, to learn the unique characteristics of the motion sequence. In contrast to the typical GCNs, our method brings a richer and more refined topological representation of skeleton data. On several large-scale benchmarks, experimental results show that the proposed model consistently surpasses the latest techniques.

## Introduction

Human motion is a key medium for understanding human behavior of robots, and human motion analysis has been widely applied in many fields, such as human–robot interaction, virtual reality, and computer animation [1–3]. In this work, we focus on the specific task of human motion forecasting that aims to estimate future human actions over a while from its historical skeleton data. Due to the unique significance in human–robot interaction and machine intelligence, it has attracted increasing attention [4–8].

Contemporary approaches regard the human skeleton as a simplified graph and then resort to graph convolutional networks (GCNs) to formulate this task, achieving impressive results [3,9–11]. These methods explicitly consider the natural structure of human poses. However, typical GCN methods can only model pairwise (2-order) connections of human joints. In real scenarios, human action often presents interactive movements of multiple joints on a specific limb, making it even more complicated, beyond the range of paired connections that GCN can represent. For instance, when dancing, the motion pattern is that the knee interacts with all the other joints on the leg to perform a graceful movement according to kinematic characteristics, and so do other limbs. This complicated high-order relationship obviously transcends the pairwise connection of the traditional GCNs [12,13].

In addition, human joints are typically driven by their parent joints to move on a spherical surface, while they cannot drive the parent’s movement. The directionality of this message passing is an essential characteristic of human action [14]. Most of the existing methods crudely treat this asymmetric relationship, which is undoubtedly unreasonable; therefore, it further deteriorates the prediction performance [9–11].

To address these limitations, we present a novel hybrid directed hypergraph convolution network (H-DHGCN), which is capable of efficiently representing the complex and diversified human skeleton. Our inspiration intuitively comes from the fact that, in natural human activities, multiple joints on each limb or the trunk always move interactively, especially the parent joint, which drives each of its child joints to perform a chain of actions. This high-order and directional correlation transcend simple pairwise connections and provide profound kinematical characteristics, which is vital and instructive for human motion prediction [15]. To encode it, we first propose to represent the human skeleton as a static directed hypergraph (S-DHG) composed of several pre-defined hyperedges, in which the degree of hyperedges may exceed 2. In contrast to standard GCNs, hypergraphs are beneficial to directly simulate the linkage of multiple joints in a practical action scenario, without indirect reasoning joint-by-joint [14,16,17]. Although the S-DHG describes the natural relationship among joints, it remains non-optimal, as the motion pattern of different actions should be theoretically depicted by different hypergraphs, while the S-DHG is fixed in the whole network update.

For this purpose, we also propose a learnable dynamic directed hypergraph (D-DHG), in which the KNN and KMeans approaches are exploited to dynamically construct the suitable topology for different samples. As a supplement to S-DHG, the proposed D-DHG improves the model flexibility, and the potential high-order directional correlation of human poses can be extracted. Finally, we integrate D-DHG and S-DHG into the hybrid form, which presents a more elaborate and richer topological representation of the 3-dimensional (3D) skeleton pose, thus facilitating motion forecasting.

Our contributions are as follows: (a) We propose to represent the human skeleton as a hypergraph to capture high-order relations. To our best knowledge, it is the first research attempt to exploit the hypergraph for human motion forecasting. (b) The proposed H-DHGCN is constructed on 2 core components: S-DHG and D-DHG, which is able to effectively access the natural topology of the human skeleton and, meanwhile, potentially capture the high-order and directional correlations. (c) Experiments clearly demonstrate that the proposed model consistently outperforms the state-of-the-art performance on 3 human action benchmarks.

The rest of this paper is organized as follows: The “Related Work” section describes the related works including human pose forecasting and hyper-graph learning. The “Methodology” section presents the proposed H-DHGCN in detail. The “Experiments” section reports the results, experiment analysis, and computation overhead with the extensive experiments. In the “Conclusion” section, we also discuss the limitations and future work of this paper.

## Related Work

### Human motion prediction

With the rapid development of deep learning technology, researchers have attempted various solutions to analyze the problem of human motion prediction [18–20]. Since human motion is essentially a sequential data, typical methods utilize the variants of recurrent neural networks (RNNs) to extract temporal patterns of motion sequences pose-by-pose [21–23]. Although a promising result has been achieved, RNN can hardly extract the structural characteristics of the human skeleton. Besides, as mentioned in previous work [24,25], due to the problem of error accumulation, RNN variants often fall into an obvious discontinuity between the first frame of the prediction and the last frame of the historical sequence, and even converge to a static pose [26–28].

Recently, as an instrumental alternative, GCNs have been proposed for forecasting human motions. Mao et al. [9] first introduce the concept of GCNs, where they consider the human pose to involve an unrestricted topology. While impressive results have been reported, because of the omission of the meaningful human skeletal structure, distorted predictions may be produced. Cui et al. [11] propose to model the pairwise connection of adjacent joints and geometrically separated joints simultaneously. Although slight improvements have been achieved, they only consider the simple 2-order relationship of the human skeleton, which violates the complex situation of multi-joint interaction. Li et al. [10] develop a novel multiscale graph to comprehensively analyze the motion sequence; however, because the asymmetric relationship between the joints is not considered, it only produces sub-par results. Li et al. [10] observe that the motion of human pose becomes more stable, and based on it, they extend [9,10] to a residual multiscale version to capture features from fine to coarse scale. Ma et al. [3] also propose to use GCNs as the basic building block, and further develop both key modules: spatial and temporal dense GCNs. Alternatively, Zhong et al. [29] present a spatial–temporal gating-adjacency GCN to encode the spatial–temporal relationship over diverse action categories, and utilize trainable adjacency to improve the generalization ability.

Despite achieving encouraging performance, GCNs are still not able to capture the high-order and directional correlations of the human skeleton. In contrast, our approach adeptly leverages the human topology with the pre-defined S-DHG, and meanwhile, with the D-DHG, the potential high-order relations among multiple joints can also be analyzed, thus extracting meaningful context for predicting human motions effectively.

### Hypergraph neural networks

It is noteworthy that, the data structure in the real world frequently involves high-order correlations between multi-objects, or even more complicated relations [29–32]. Compared with GCNs in which the degree of all edges is fixed to 2, a hypergraph can utilize hyperedges with a liberalized degree to express this higher-order interaction (beyond pairwise connections). If all edges in a hypergraph contain only 2 vertices, the hypergraph will degenerate into an ordinary graph. Therefore, the hypergraph is essentially a generalization of the ordinary graph, which can theoretically achieve better performance than the traditional GCN algorithms.

Feng et al. [12] propose a general hypergraph neural network, which is then applied to citation network classification, achieving remarkable results. Zhang et al. [33] introduce a self-attention hypergraph representation learning model to the task of outsider identification, which obtains significant improvements. Tran et al. [17] present a directed hypergraph algorithm into page ranking. It considers the directionality of information transfer among nodes on the hyperedge. Our motivation is partly inspired by the above literature. Human activities exhibit high-order interactions and pregnant asymmetries among multiple joints, which are of particular significance in understanding the motion pattern. Our model focuses on these characteristics to extract a refined and informative semantic, so as to obtain high-quality generation.

## Methodology

### Problem statement and definition

Let *X*_−*T* + 1 : 0_ = [*X*_−*T* + 1_…, *X*_−1_, *X*_0_] ∈ *R*^*T* × *N* × *D*^ be the historical human poses, where *X_t_* ∈ *R*^*N* × *D*^ expresses a frame at a certain timestamp with *N* joints and *D* = 3 dimensions. Then, the predicted actions are defined as $Y_{1:\Delta T}=\left[{\hat{Y}}_{1},,,\ldots ,,,{\hat{Y}}_{\Delta T-1},,,{\hat{Y}}_{\Delta T}\right]$, and formally, *Y*_1 : Δ*T*_ = [*Y*_1_, …, *Y*_Δ*T* − 1_, *Y*_Δ*T*_] denotes the corresponding ground truth. For 3D skeleton data, the high-order and directional relationships between joints reflect the vital and instructive motion patterns. To accurately capture these meaningful connections, we propose a hybrid directed hypergraph convolution to learn a refined mapping: $M:X_{-T:0}\to Y_{1:\Delta T}$, to make *Y*_1 : Δ*T*_ as close to *Y*_1 : Δ*T*_ as possible.

Concretely, the proposed approach mainly involves the following 2 components: S-DHG and D-DHG, to learn the specific high-order topology and the potential one, respectively. Next, we will illustrate the details.

### Static directed hypergraph

**Fig. 1.**
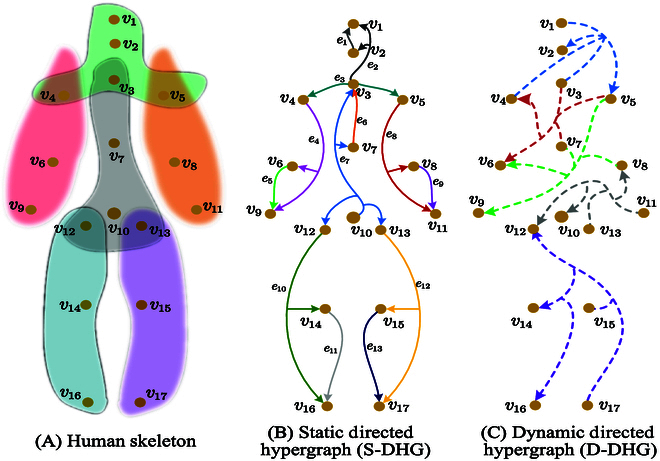
(A) Human skeleton, divided into 6 parts: a head, a trunk, 2 arms, and 2 legs. (B) Static directed hypergraph (S-DHG). *e_ϵ_* is the hyperedge formed by a single head and many tails (possibly one to one). (C) Dynamic directed hypergraph (D-DHG), where the dotted line denotes the dynamic hyperedge. It can be established adaptively according to the motion pattern of the specific sequence.

In the context of human movements, all joints on the limbs or trunk are pulled to move together, which involves an explicit direction from the parent joint to the child joint. For example, when walking, the joints of the legs operate interactively in the form of the hip driving the knee, and then the knee driving the ankle, which is similar to the movement of the joints in the arms. This intuitive observation inspires us to naturally divide the human skeleton into 6 parts, i.e., 2 arms, 2 legs, 1 torso, and 1 head, as shown in Fig. 1. To capture the complex relationship across multiple human joints, we naturally divide the human skeleton into 6 intersecting subsets, i.e., 2 arms, 2 legs, 1 torso, and 1 head, as shown in Fig. 1A. Within each part, we establish several directed hyperedges. The direction of each hyperedge is from the parent joints to their child joints to model the directionality. However, these parts are not separate but transmit messages through their intersections. Because a parent affects all its tail joints, in each directed hyperedge, the number of the head is fixed to 1, and the tail nodes are all its child joints. Figure 1B provides the specific construction of the S-DHG.

Based on the above illustration, we represent the human pose as a directed hypergraph $H=\left(V,,,E,,,W\right)$. V is the set of *N* vertices. E is the set of *M* hyperedges, where the degree of each edge can be greater than 2 to construct high-order correlations. The diagonal matrix *W* =  *diag*(*w*_1_, *w*_2_, …, *w_M_*) ∈ *R*^*M* × *M*^ assigns the weights to each edge. Note that we re-express each hyperedge e∈E as *e* ∈ (*e^head^*, *e^tail^*). *e^head^* and *e^tail^* are called the head and tail of the hyperedge *e*. *e^head^* ∪ *e^tail^* denotes the vertices on edge *e*. For each directed hyperedge *e*, we notice that $e^{\text{head}}\neq Ø$, $e^{\text{tail}}\neq Ø$, and *e^head^* ∩ *e^tail^* = ⌀.

Instead of the adjacency matrix of GCNs, the directed hypergraph can be expressed as 2 incidence matrices, denoted as *H^tail^* and *H^head^*, where *H^tail^* represents the connections with the tail and *H^head^* represents the connections with the head. As shown in Fig. 1B, when a vertex *v_i_* is connected by the head of a hyperedge $e_{\epsilon }^{\text{head}}$, then $H_{i,\epsilon }^{\text{head}}=1$; on the contrary, when it is connected by the tail of the hyperedge $e_{\epsilon }^{\text{tail}}$, $H_{i,\epsilon }^{\text{tail}}=1$. Mathematically, it can be formulated as:

$$H_{i,\epsilon }^{\text{head}}=\left\{\begin{array}{c} 1,\;\text{if}\;v_{i}\in e_{\epsilon }^{\text{head}}\\ 0,\;\text{otherwise} \end{array}\right.$$ (1)

$$H_{i,\epsilon }^{\text{tail}}=\left\{\begin{array}{c} 1,\;\text{if}\;v_{i}\in e_{\epsilon }^{\text{tail}}\\ 0,\;\text{otherwise} \end{array}\right.$$ (2)

The main principle of defining convolution on a directed hypergraph is that propagation should be performed along the information transfer direction of those vertices on the hyperedge. To this end, inspired by [17], we directly develop the hypergraph convolution on the S-DHG, called static directed hypergraph convolution network (S-DHGCN), expressed as:

$$x_{i}^{\left(l+1\right)}=\sigma \left(\sum_{j=1}^{N}‍\sum_{\epsilon =1}^{M}‍H_{i,\epsilon }^{\text{head}}H_{j,\epsilon }^{\text{tail}}W_{\epsilon ,\epsilon }x_{j}^{\left(l\right)}\Theta \right),$$ (3)

where $x_{i}^{\left(l+1\right)}$ is the output feature, $x_{j}^{\left(l\right)}$ is the latent code of the vertex *v_j_* at *l*th layer. *σ*(·) is the activation function, e.g., *Mish* [34], and Θ ∈ *R*^*C*^(*l*)^ × *C*^(*l* + 1)^^ is the learnable parameter. Equation 3 indicates that the message is passed from the single parent joint to its child joints.

The above S-DHGCN can be simplified into a matrix form:

$$X^{\left(l+1\right)}=\sigma \left(H^{\text{head}}W{H^{\text{tail}}}^{T}X^{\left(l\right)}\Theta \right),$$ (4)

where *H^head^* ∈ *R*^*N* × *M*^, *H^tail^* ∈ *R*^*N* × *M*^ are the incidence matrices. Besides, *X*^(*l*)^ ∈ *R*^*N* × *C*^(*l*)^^ and *X*^(*l* + 1)^ ∈ *R*^*N* × *C*^(*l* + 1)^^ are the input and output of the *l-*th layer.

When we stack multiple Eq. 4, it may lead to significant training instability. To solve this, we propose to utilize asymmetric normalization for the incidence matrices. Assuming $D_{v}^{\text{head}}\in R^{N,N}$ and $D_{e}^{\text{tail}}\in R^{M,M}$ are the degree matrix of the head for the vertex *v* and the tail for the hyperedge *e* respectively, it can be then expressed as:

$$D_{v}^{\text{head}}\left(i,,,i\right)=\sum_{\epsilon =0}^{M}‍W_{\epsilon ,\epsilon }H^{\text{head}}\left(i,,,\epsilon \right),$$ (5)

$$D_{e}^{\text{tail}}\left(\epsilon ,,,\epsilon \right)=\sum_{i=0}^{N}‍H^{\text{tail}}\left(i,,,\epsilon \right).$$ (6)

Then, the normalized form of S-DHGCN is obtained:

$$X^{\left(l+1\right)}=\sigma \left({D_{v}^{\text{head}}}^{-1}{H^{\text{head}}}^{T}W{D_{e}^{\text{tail}}}^{-1}H^{\text{tail}}X^{\left(l\right)}\Theta \right),$$ (7)

where the ${D_{v}^{\text{head}}}^{-1}{H^{\text{head}}}^{T}W{D_{e}^{\text{tail}}}^{-1}H^{\text{tail}}$
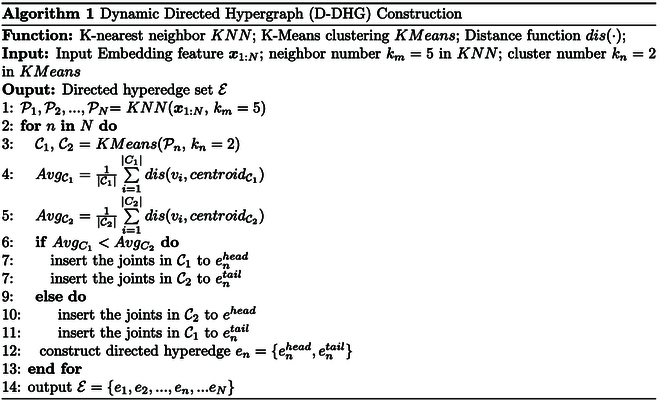
 is the directed hypergraph Laplacian. With the above operation, the hyperedge state is propagated from the vertices connecting it, and the tail nodes receive the information from the head joints. Then, the high-order and directional relationships of the human skeleton can be effectively captured in end-to-end training via a gradient-based optimizer.

For the sake of simplicity, we denote the subscript *S* as the above S-DHG, and *W* =  *diag* (1, 1, …, 1). Let ImpShead=Dvhead−1HheadT be the convolution operator of head vertices updating in S-DHG, and ImpStail=Detail−1Htail be the corresponding one of tail vertices. Finally, Eq. 7 can be re-written as the following formula:

Xl+1=σImpSheadImpStailXlΘ. (8)

Through the operators ImpShead and ImpStail, the proposed S-DHG structure is pre-defined and frozen in the whole network updating. Therefore, the S-DHGCN gains insight into the natural topology, and captures meaningful high-order and directional information.

### Dynamic directed hypergraph

Nevertheless, the S-DHG structure remains non-optimal. The major reason is that the motion patterns of different actions should be depicted by different structures. In S-DHG, the number of hyperedges and the nodes on each hyperedge are typically unchanged; hence, it is challenging to adaptively extract the dynamic correlation of complex actions. To solve it, we propose the novel D-DHG to adaptively describe the high-order structure of motion sequences.

Specifically, we construct the D-DHG using the following 2 steps: (a) Intuitively, in embedding space, the closer the distance of 2 nodes, the higher the correlation, and it is more reasonable to connect them to the same hyperedge. Therefore, for the feature ***x****_i_* of each human joint, we calculate its Euclidean distance *dis*(·) from the other joints *x_j_*, and connect the *k_m_* − 1 smallest distances and *v_i_* to the same hyperedge using *KNN*. Then, we obtain *N* hyperedges, each of which contains *k_m_* nodes. Note that we denote the node set on each hyperedge as $P_{n}$. (b) We then exploit *KMeans* on each $P_{n}$, and until convergence, the 2 disjoint subsets $C_{1}$, $C_{2}$ are generated, where their centroids are denoted as $\text{centroi}d_{C_{1}},\text{centroi}d_{C_{2}}$. Then, for $C_{1}$, $C_{2}$, the one with a smaller average distance from all joints to the centroid is connected to *e^head^*, and the other is the tail joints *e^tail^*. Finally, the dynamic directed hyperedge $e_{n}=\left\{e_{n}^{\text{head}},,,e_{n}^{\text{tail}}\right\}$ is established. The overall procedure of the D-DHG construction is described in Algorithm 1.

With the above algorithm, the proposed D-DHG is adaptively constructed, in which each directed hyperedge contains *k_m_* joints (involving *k_n_* = 2 categories w.r.t. head or tail joint) to enhance the flexibility. Therefore, the motion patterns of various activities can be considered.

We then develop the dynamic directed hypergraph convolution network (D-DHGCN) on the D-DHG. Similar to the S-DHGCN, the update of the D-DHGCN can be directly expressed as:

Xl+1=σImpDheadImpDtailXlΘ, (9)

where ImpDhead is the convolution operator of head nodes in D-DHG, and the ImpDtail is one of the tail nodes.

### Implementation details

**Fig. 2.**
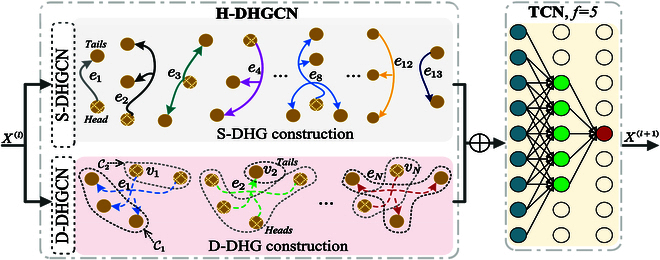
Illustration of our temporal–spatial block consisting of the proposed H-DHGCN and TCN, where the H-DHGCN is formed by adding the output of the S-DHGCN and D-DHGCN for extracting both the high-order human topology and the semantic directionality. In the S-DHGCN, the hypergraph points from the head to tails, in the form of one to many, whereas in the D-DHGCN, it is many to many, constructed dynamically. Note that there are *N* edges in D-DHGCN, each of which contains *k_m_* = 5 joints calculated by KNN. After that, KMeans is used to categorize these nodes into *k_n_* = 2 clusters, where the solid circles are the head nodes, and the hollow circles are the tail nodes.

In the development of our proposed model, we leverage the strengths of 2 key components, namely, the S-DHGCN and D-DHGCN. These 2 components are strategically stacked to form the H-DHGCN, a novel architecture that takes into account both the specific human topology and the implicit high-order relationships within human poses. We note that the H-DHGCN is dynamically constructed, and the number of hyperedges and the nodes on each hyperedge are not fixed. Therefore, it is capable of adaptively capturing the high-order and directional correlations of human poses. Moreover, the D-DHGCN is achieved by Kmeans clustering (*K_n_* = 2) and KNN (*K_m_* = 5), which means that each hyperedge contains 1 head node and 4 tail nodes. To capture the temporal dynamics inherent in motion sequences, we employ temporal convolutional networks (TCNs). The TCN layers are configured with a filter size of *f* = 5 × 1, as visually depicted in Fig. 2. Our proposed model consists of a total of 9 residual blocks, each comprising an H-DHGCN layer and a TCN layer, both followed by batch normalization (BN). This design is aimed at extracting rich and refined representations from human action sequences. Additionally, 2 extra H-DHGCN layers are appended to the beginning and end of the network to enhance its overall effectiveness. Within each residual block, the filter size of the H-DHGCN layer is set to Θ ∈ *ℝ*^512 × 512^. Both the H-DHGCN and TCN layers are activated using the Mish function [34], incorporating a dropout rate of 0.25 to enhance model generalization. For training our model, we employ the average *L*_2_ distance as the loss function, consistent with previous works such as [9,35]. The predicted result ${\hat{Y}}_{1:\Delta T}$ is compared against the ground truth **Y**_1:Δ*T*_. The optimization process is facilitated using the Adam optimizer [36], and we initialize the learning rate at 0.01. A decay rate of 0.98 is applied every 2 epochs to facilitate convergence. It is important to note that our model is trained across all motion categories, ensuring the development of an action-agnostic model. The entire implementation is carried out using the PyTorch framework, providing a robust and scalable environment for model development and experimentation.

## Experiments

### Datasets and experimental settings

#### Datasets

We evaluate on 3 motion capture datasets, Human3.6M, CMU MoCap, and 3DPW MoCap. Human3.6M [37] includes 15 motion categories, specifically including walking, eating, smoking, discussion, direction, greeting, phoning, posing, purchases, sitting, sitting down, taking photo, waiting, walking dog, and walking together, performed by 7 actors. We also note that the Human3.6M dataset contains >1,000 min of motion capture data, which is the largest publicly available dataset for human motion prediction. Specifically, each pose *X_i_* ∈ *R*^17 × 3^ is represented using the position of 17 joints in Cartesian space. In our experiments, all the sequences are downsampled to 25 fps. Consistent with [38,39], we select the action of subject-5 (S5) as the test sample, S11 as the validation, and then the rest is for training. In CMU MoCap [40], 8 action categories (basketball, basketball signal, directing traffic, jumping, running, soccer, walking, and wash window) are selected to report the result, where the validation set is unavailable and the test/train sets are consistent with the previous paper [11,41]. Other preprocessing solutions are the same as for the Human3.6M dataset. We also report the experimental results on the 3DPW Mocap Dataset [42], where the officially recommended training, validation, and testing set are exploited. The 3DPW MoCap dataset contains 60 video sequences (372 min) performed by 18 actors, each of which is annotated with 3D human poses, but does not explicitly categorize the action type. We also note that the Human3.6M dataset is captured in a controlled environment, the CMU MoCap dataset is captured in an unconstrained environment, while the 3DPW dataset is captured in both indoor and outdoor scenario. The proposed model and the baseline methods are evaluated on the 3 datasets, which covers different aspects and complements each other, and can more comprehensively verify the effectiveness of our model.

#### Metrics

In our model, both the input and output are 3D coordinate-based skeleton data. Therefore, for position-based sequences, we first use the mean per joint position error (MPJPE) [37] to report the 3D error in millimeters. Moreover, the predicted position is also converted into an Euler angle, and then the angle error of the final angle-based prediction is evaluated using mean angle error (MAE). Besides, the predicted pose is also animated to investigate the qualitative performance.

**Baselines.** We select the following baselines to evaluate the performance of our model: dynamic multi-scale graph neural network (DMGNN) [10], learning trajectory dependency (LTD) [9], multi-scale residual graph convolution network (MSR) [43], and gradually generating better initial guess (PGBIG) [3]. DMGNN [10] exploits multi-scale GCNs to capture the spatial information of the motion sequences. LTD [9] converts the motion sequence to the frequency domain and then resorts to fully connected GCN to predict future human actions. MSR [43] extends LTD to extract multi-scale features. PGBIG [3] generates a better initial guess of the final target future pose to obtain the high-quality future motions. For a fair comparison, our model is compared with their re-trained models using the released code the published results in their paper.

### Comparisons on the Human3.6M dataset

Following the previous work [10,11], we first visualize the character animation of each time step for qualitative comparison. Besides, MPJPE and MAE evaluation criteria are used for quantitative comparison, in which the angle error is calculated by transforming the predicted position-based sequence into angle space, while the 3D error is calculated directly. In all experiments, the length of the observed and predicted poses is 1,000 ms.

**Fig. 3.**
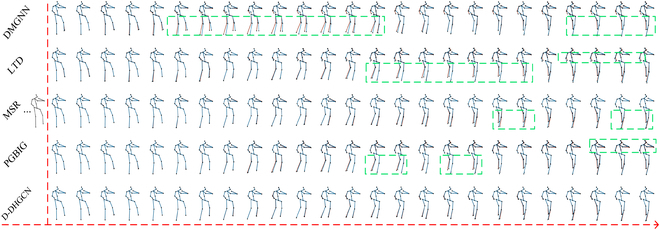
Qualitative comparison of the predicted human poses under phoning activity. Predicted human poses (with an interval of 40 ms) from 40 ms to 1,000 ms are shown. In each row, the underlying red skeletons are the ground truth, and the blue ones are the predicted results. Note that the green boxes highlight the predicted unreasonable segments that are visually more distinct from the ground truth, in which the green boxes highlight the unreasonable segments. From top to bottom, we show the result of the DMGNN [10], LTD [9], MSR [43], PGBIG [3] and the proposed H-DHGCN. From the generated animations, we observe that the proposed H-DHGCN produces a more realistic visualization in almost all scenarios.

As shown in Fig. 3, we visualize the animation at each timestamp on the *phoning* activity from the Human3.6M dataset, in which the red dotted line distinguishes between observations and predictions. From top to bottom, we show the GT, and the generation of the GCN-based method, i.e., LTD [9], DMGNN [10], MSR [43], and PGBIG [3], and the proposed H-DHGCN. The green dotted rectangles indicate unreasonable segments. For short-term prediction (first 10 predicted frames), we observe that the baselines and our approach have almost achieved indistinguishable results. However, the visualization of the proposed model is still slightly superior to that of the competitive methods. It is worth noting that, with the extension of the predicted range, the superiority of the proposed H-DHGCN gradually appears. In particular, we observe that the refinement of the legs and arms obtained by our H-DHGCN is higher in almost all scenarios, which is more matched to the GT. The above discussion evidences our remarkable visualization.

Now, we quantitatively compare our approach with all the baselines in terms of MAE measures. Table 1 presents the numerical results on 4 representative activities. Specifically, we report the result at 80 ms, 160 ms, 320 ms, and 400 ms for short-term prediction, and then the predicted pose of 1,000 ms is calculated as the long-term prediction. Generally speaking, our method achieves better accuracy, regardless of short-range or long-range predictions. Actually, such a small error is hardly detected by human eyes in character animations, which also confirms the excellent performance in qualitative results in the above paragraph.

**Table 1.** Comparisons of MAE on 4 representative activities from the Human3.6M dataset, which are calculated by converting the predicted position into angle space. The best result is in boldface, and the second is underlined.

Motion	Walking				Eating				Smoking				Discussion			
Milliseconds	80	160	400	1,000	80	160	400	1,000	80	160	400	1,000	80	160	400	1,000
DMGNN [10]	0.18	0.31	0.58	0.75	0.17	0.30	0.59	1.14	0.21	0.39	0.77	1.52	0.26	0.65	0.99	1.45
LTD [9]	0.18	0.31	0.56	0.79	0.16	0.29	0.62	**1.05**	0.22	0.41	0.80	1.23	0.20	0.77	0.85	1.57
MSR [43]	0.16	0.29	0.57	0.71	0.16	0.27	0.64	1.17	**0.20**	0.38	0.82	1.17	**0.19**	0.53	0.83	1.55
PGBIG [3]	0.18	0.31	**0.49**	0.79	0.16	0.29	**0.50**	**1.05**	0.22	0.41	0.86	1.23	0.20	0.51	0.85	1.57
H-DHGCN	**0.15**	**0.27**	0.50	**0.67**	**0.15**	**0.25**	0.57	1.09	**0.20**	**0.36**	**0.75**	**1.16**	**0.19**	**0.49**	**0.81**	**1.42**

Compared with the typical recurrent models [19,21], GCNs are able to establish the spatial dependency of human joints explicitly. Although promising results have been achieved, human motion is a complex natural manifestation in which human joints show significant high-order correlation, whereas GCN-based approaches can only capture pairwise connections but have a low capability for high-order relations. Therefore, corresponding to Table 1, those GCN-based methods only achieve subpar results. By contrast, our H-DHGCN relies on both the S-DHG and D-DHG, which efficiently extracts complex high-order dependencies of multi-joints and dynamically analyzes the potential human structure of different motion sequences. Moreover, it also considers the important asymmetric interaction among joints, which leads to better performance.

Next, we follow the previous work [9,11], and directly measure themean 3D error on the predicted 3D coordinate. As shown in Table 2, it provides a detailed comparison under a total of 15 activities on the Human3.6M dataset. From the results, we observe that the proposed H-DHGCN has almost consistently achieved superiority in almost all scenarios, even compared with the state-of-the-arts. Although GCN-based baselines are capable of accessing the topology of the human skeleton, they can only establish the 2-order relationship of the joint pair; hence, inaccurate results may be obtained. As a comparison, our approach captures the complex high-order correlation and directional connection of human joints simultaneously, thus yielding a higher-quality prediction.

**Table 2.** MPJPE comparison for per action on the total of 15 activities of the Human3.6M dataset. The state-of-the-art result is highlighted in boldface, and the second is underlined.

	Walking					Eating					Smoking				
Milliseconds	80	160	320	400	1,000	80	160	320	400	1,000	80	160	320	400	1,000
DMGNN [10]	17.3	30.7	54.6	65.2	95.8	11.0	21.4	36.2	43.9	86.7	9.0	17.6	32.1	40.3	72.2
LTD [9]	12.3	23.0	39.8	46.1	59.8	8.4	16.9	33.2	40.7	77.8	7.9	16.2	31.9	38.9	72.6
MSR [43]	12.2	22.7	38.6	45.2	63.0	8.4	17.1	33.0	40.4	77.1	8.0	16.3	31.3	38.2	71.6
PGBIG [3]	10.2	19.8	34.5	40.3	56.4	**7.0**	15.1	**30.6**	**38.1**	76.0	**6.6**	14.1	28.2	34.7	69.5
H-DHGCN	**9.6**	**17.7**	**33.6**	**38.6**	**53.1**	7.2	**14.7**	31.1	38.7	**73.3**	6.9	**13.8**	**26.1**	**33.1**	**64.4**
	Discussion					Directions					Greeting				
Milliseconds	80	160	320	400	1,000	80	160	320	400	1,000	80	160	320	400	1,000
DMGNN [10]	17.3	34.8	61.0	69.8	138.3	13.1	24.6	64.7	81.9	115.8	23.3	50.3	107.3	132.1	157.7
LTD [9]	12.5	27.4	58.5	71.7	121.5	9.0	19.9	43.4	53.7	101.8	18.7	38.7	77.7	93.4	148.8
MSR [43]	12.0	26.8	57.1	69.7	117.6	8.6	19.7	43.3	53.8	100.6	16.5	37.0	77.3	93.4	147.2
PGBIG [3]	10.0	23.8	53.6	**66.7**	118.2	**7.2**	**17.6**	40.9	51.5	**100.4**	15.2	**34.1**	71.6	87.1	143.5
H-DHGCN	**9.1**	**21.5**	**53.2**	68.5	**112.3**	7.7	20.2	**40.6**	**50.5**	100.6	**14.6**	35.7	**67.8**	**85.7**	**141.5**
	Phoning					Posing					Purchase				
Milliseconds	80	160	320	400	1,000	80	160	320	400	1,000	80	160	320	400	1,000
DMGNN [10]	12.5	25.8	48.1	58.3	98.6	15.3	29.3	71.5	96.7	310.1	21.4	38.7	75.7	92.7	153.8
LTD [9]	10.2	21.0	42.5	52.3	103.1	13.7	29.9	66.6	84.1	173.0	15.6	32.8	65.7	79.3	143.5
MSR [43]	10.1	20.7	41.5	51.3	104.4	12.8	29.4	67.0	85.0	174.3	14.8	32.4	66.1	79.6	139.2
PGBIG [3]	**8.3**	**18.3**	38.7	48.4	102.7	10.7	25.7	60.0	76.6	164.8	12.5	**28.7**	60.1	**73.3**	133.3
H-DHGCN	9.7	18.8	**36.4**	**45.7**	**102.2**	**10.5**	**23.5**	**57.4**	**72.2**	**160.8**	**12.1**	30.3	**58.3**	74.2	**129.2**
	Sitting					Sitting down					Taking photo				
Milliseconds	80	160	320	400	1,000	80	160	320	400	1,000	80	160	320	400	1,000
DMGNN [10]	11.9	25.1	44.6	50.2	104.9	16.1	31.1	61.5	75.5	150.2	9.9	20.9	45.0	56.6	119.8
LTD [9]	10.6	21.9	46.3	57.9	119.7	15.0	32.9	77.1	93.0	168.8	13.6	29.0	46.0	58.8	120.7
MSR [43]	10.5	22.0	46.3	57.8	120.0	16.1	31.6	62.5	76.8	155.5	9.9	21.0	44.6	56.3	121.9
PGBIG [3]	**8.8**	**19.2**	42.4	53.8	116.1	13.9	27.9	**57.4**	**71.5**	147.8	8.4	18.9	42.0	53.3	118.6
H-DHGCN	8.9	21.6	**40.8**	**51.3**	**112.4**	**12.8**	**25.4**	64.7	72.9	**144.3**	**7.5**	**17.1**	**40.3**	**52.6**	**114.6**
	Waiting					Walking dog					Walking together				
Milliseconds	80	160	320	400	1,000	80	160	320	400	1,000	80	160	320	400	1,000
DMGNN [10]	11.4	24.0	50.1	61.5	108.1	23.4	46.2	83.5	96.0	148.9	14.3	26.7	50.1	63.2	65.6
LTD [9]	12.2	24.2	59.6	77.5	136.7	47.1	93.3	160.1	171.2	182.3	10.5	21.0	38.5	45.2	115.9
MSR [43]	10.7	23.1	48.3	59.2	106.3	20.7	42.9	80.4	93.3	148.2	10.6	20.9	37.4	43.9	65.9
PGBIG [3]	**8.9**	20.1	**43.6**	54.3	103.4	**18.8**	39.3	73.7	86.4	139.8	8.7	18.6	34.4	41.0	64.3
H-DHGCN	**8.9**	**19.6**	44.4	**52.2**	**103.2**	20.3	**38.2**	**72.5**	**83.2**	**137.4**	**8.5**	**17.6**	**33.8**	**40.2**	**62.7**

### Results on the CMU and 3DPW MoCap datasets

To fully investigate the proposed model, consistent with [3,31], we also report the 3D error of the MPJPE metric under both the CMU and 3DPW MoCap datasets, as shown in Tables 3 and 4. From the results, we observe that our approach widely outperforms the competitors, regardless of short-term or long-term prediction. These empirical experiments evidence that our approach is excellent in predicting future actions again.

**Table 3.** MPJPE comparison per action on 8 activities of CMU MoCap. The best result is highlighted in boldface, and the second is underlined.

	Basketball					Basketball signal					Directing traffic				
Milliseconds (ms)	80	160	320	400	1,000	80	160	320	400	1,000	80	160	320	400	1,000
DMGNN [10]	15.6	28.7	59.0	73.1	138.6	5.0	9.3	20.2	26.2	52.0	10.2	20.9	41.6	52.3	111.2
LTD [9]	11.7	21.3	41.0	50.8	98.0	3.3	6.3	13.6	18.0	54.0	6.9	13.7	30.3	40.0	114.2
MSR [43]	10.3	18.9	37.7	47.0	87.0	3.0	5.7	12.4	16.3	47.9	5.9	12.1	28.4	38.0	111.0
PGBIG [3]	**9.5**	17.5	35.3	44.2	84.1	**2.7**	4.9	10.8	14.6	50.2	**4.8**	**9.8**	23.6	32.3	102.3
H-DHGCN	10.2	**17.4**	**34.8**	**43.7**	**83.4**	3.1	**5.1**	**10.7**	**14.0**	**47.8**	5.5	10.7	**21.4**	**31.6**	**102.1**
	Jumping					Running					Soccer				
Milliseconds (ms)	80	160	320	400	1,000	80	160	320	400	1,000	80	160	320	400	1,000
DMGNN [10]	32.0	54.3	96.7	119.9	224.6	14.9	25.3	52.2	65.4	111.9	9.6	15.5	26.0	30.4	67.0
LTD [9]	17.2	32.4	60.1	72.6	127.4	13.3	24.0	43.8	53.2	108.3	6.6	10.7	17.4	20.4	34.4
MSR [43]	15.0	28.7	55.9	69.1	124.8	**10.9**	**19.5**	**37.1**	46.4	99.3	6.3	**10.3**	17.6	21.1	39.7
PGBIG [3]	13.9	**27.8**	55.8	69.0	125.6	11.1	20.6	39.5	48.7	99.0	**6.2**	**10.3**	16.8	**19.8**	33.9
H-DHGCN	**11.7**	28.4	**54.7**	**68.3**	**124.5**	11.3	20.4	37.5	**45.6**	**96.4**	**6.2**	10.6	**16.7**	20.7	**33.5**
	Walking					Wash window									
Milliseconds (ms)	80	160	320	400	1,000	80	160	320	400	1,000					
DMGNN [10]	7.9	14.7	33.3	44.2	82.8	13.6	24.1	47.0	58.8	112.6					
LTD [9]	6.0	11.6	24.8	31.6	67.0	9.3	17.1	33.0	40.9	86.2					
MSR [43]	5.5	11.1	25.1	32.5	71.3	8.1	15.2	30.6	38.6	83.0					
PGBIG [3]	**4.6**	9.2	20.9	27.3	65.7	**7.6**	14.3	29.0	36.6	80.1					
H-DHGCN	4.7	**9.1**	**19.4**	**26.7**	**65.2**	7.7	**13.5**	**26.3**	**35.8**	**77.3**					

**Table 4.** Mean 3D error on the 3DPW Mocap dataset. The best result is highlighted in boldface, and the second is underlined.

Milliseconds (ms)	200	400	600	800	1,000
DMGNN [10]	37.3	67.8	94.5	109.7	123.6
LTD [9]	35.6	67.8	90.6	106.9	117.8
MSR [31]	37.8	71.3	93.9	110.8	121.5
PGBIG [3]	29.3	58.3	79.8	94.4	104.1
H-DHGCN	**28.4**	**54.7**	**78.1**	**91.8**	**101.0**

### Efficiency analysis

To evaluate the efficiency of our proposed H-DHGCN, we compare it with the latest approaches in terms of consuming time and parameter number of the long-term prediction (1,000 ms) on the Human3.6M dataset. The results are shown in Table 5. From the result, we observe that our method has a smaller parameter number, mainly because the hypergraph convolution is able to directly analyze the high-order spatial correlation among multiple joints, surpassing the 2 nodes in the standard graph convolution. On the other hand, we note that the proposed H-DHGCN achieves the second-rank running time, which is slightly slower than the state-of-the-art LTD [9]. All experiments are implemented on a single NVIDIA GeForce RTX 3090Ti GPU.

**Table 5.** Analysis of the number of parameters and time overhead of different methods. The best result is highlighted in boldface, and the second is underlined.

Methods	DMGNN [10]	LTD [9]	MSR [43]	PGBIG [3]	H-DHGCN (Ours)
No. of parameters	4.01M	2.56M	6.30M	4.72M	**2.43M**
Time overhead	134 ms	**97 ms**	152 ms	122 ms	113 ms

### Ablation experiments

To study the effect of various aspects, we run ablation studies to analyze our approach, which measures the average MPJPE in the Human3.6M dataset.

1. Different representations of the human skeleton. In our model, we exploit 2 hypergraph structures to represent the human pose, including S-DHG, and D-DHG. Moreover, we perform the hypergraph convolution on them (S-DHGCN and, D-DHGCN) to consider both the natural human topology and the potential high-order directed correlation. To verify the effectiveness of our S-DHGCN and D-DHGCN, we study the effects of retaining one of them respectively. As reported in Table 6, we observe that the D-DHGCN brings more improvements than the S-DHGCN, and when both are introduced concurrently, a better result is achieved. Therefore, we reasonably conclude that simultaneous modeling of the specific high-order connections and the potential relations, is beneficial for motion forecasting.

**Table 6.** Impact of different hypergraph structures. The best result is highlighted in boldface, and the second is underlined.

S-DHGCN	D-DHGCN	80 ms	160 ms	320 ms	400 ms	1,000 ms
√	×	11.23	25.95	54.45	60.12	115.27
×	√	11.35	22.19	50.21	59.50	114.23
√	√	**10.29**	**20.95**	**46.73**	**57.31**	**107.47**

2. Standard hypergraph vs. directed hypergraph. In this work, our inspiration comes from the fact that human joints are typically activated by the parent joint, showing the explicitly directed message passing. To verify it, we run the ablation studies, in which both the proposed S-DHGCN and D-DHGCN are replaced by their standard (undirected) versions. Note that, except for expressing the human body as the undirected hypergraphs, other components remain unchanged. From Table 7, we observe that our directed hypergraph achieves a lower error, which evidences that the motion pattern of human joints involves directional information, which can be well captured by the directed hypergraphs.

**Table 7.** Standard hypergraph vs. directed hypergraph. The best result is highlighted in boldface.

Hypergraph	80 ms	160 ms	320 ms	400 ms	1,000 ms
Undirected	14.52	27.93	53.30	66.17	118.26
Directed	**10.29**	**20.95**	**46.73**	**57.31**	**107.47**

3. Effect of different *k_m_* in KNN. We utilize the KNN method to construct the D-DHG, where the number of human joints in each hyperedge is determined by *k_m_*. Moreover, the KMeans (with *k_n_* = 2) is used to select the head and tail joints in the directed hypergraph. Because the cluster number of the KMeans is fixed *k_n_* = 2, in this part, we only set different values for *k_m_* to conduct comparative experiments to find its best configuration. As shown in Table 8, we observe that when *k_m_* < 5, the performance begins to decline. The influence of *k_m_* of D-DHGCN proves that there is a threshold for the joint number constituting a directed hyperedge. Notably, *k_m_* = 5 achieves a better result, and the larger value brings no benefits.

**Table 8.** Impact with different *k_m_* in our KNN. The best result is highlighted in boldface, and the second is underlined.

KNNs	80 ms	160 ms	320 ms	400 ms	1,000 ms
*k_m_* = 3	12.25	24.73	53.21	65.50	121.13
*k_m_* = 4	12.54	22.39	50.11	63.15	120.03
*k_m_* = 5	**10.29**	**20.95**	**46.73**	**57.31**	**107.47**
*k_m_* = 6	11.02	22.54	49.26	61.50	110.73

4. Effect of different filter size *f* of TCNs. We exploit TCNs to extract the temporal correlation of inter-frames. To verify the influence of filter size, we chose *f* = {3, 5, 7}. From able 9, we observe that when *f* = 5, a balance is achieved between long-range and local temporal correlation, which, accordingly, generates a better generation.

**Table 9.** Impact with different *k_m_* in our KNN. The best result is highlighted in boldface, and the second is underlined.

Filter size	80 ms	160 ms	320 ms	400 ms	1,000 ms
*f* = 3	12.46	27.78	53.23	69.35	113.65
*f* = 5	**10.29**	**20.95**	**46.73**	57.31	**107.47**
*f* = 7	11.26	24.35	50.28	**57.11**	109.49

## Conclusion

In this work, we have proposed a novel H-DHGCN for predicting future human motions from its historical observations. To achieve it, we construct 2 hypergraph structures of the human skeleton—S-DHG and D-DHG—to consider the specific and potential high-order correlations. In contrast to simplistic GCNs, our model flexibly extracts complex patterns of 3D skeleton-based poses and then establishes meaningful semantics. Moreover, with empirical experiments, we verify that the asymmetric (directional) relationship is conducive to human motion modeling as well as the forecasting. Moreover, we demonstrate that the proposed H-DHGCN significantly exceeds the state-of-the-art approaches, regardless of short-horizon or long-horizon prediction. Our code will be publicly available.

Despite the promising results achieved by our H-DHGCN, there is still room for improvement in the future. For example, the potential of the hypergraph convolution in capturing the long-term correlation of human sequences needs to be explored. Moreover, we will consider the possibility of reducing the computational cost of the dynamic hypergraph construction, which is conducive to the real-time application of our model.

## Data Availability

We used three datasets, all of which are publicly accessible. Human3.6M: http://vision.imar.ro/human3.6m/description.phpCMU; MoCap: http://mocap.cs.cmu.edu/; 3DPW: https://virtualhumans.mpi-inf.mpg.de/3DPW/

## References

[1] Liu M, Meng F, Liang Y. Generalized pose decoupled network for unsupervised 3d skeleton sequence-based action representation learning. Cyborg Bion Syst. 2022;2022:0002. 10.34133/cbsystems.0002 PMC10076048 37040281

[2] Gao Q, Deng Z, Ju Z, Zhang T. Dual-hand motion capture by using biological inspiration for bionic bimanual robot teleoperation. Cyborg Bion Syst. 2023;4:0052. 10.34133/cbsystems.0052 PMC10499487 37711160

[3] Ma T, Nie Y, Long C, Zhang Q, Li G. Progressively generating better initial guesses towards next stages for high-quality human motion prediction. In: *Proceedings of the IEEE/CVF Conference on Computer Vision and Pattern Recognition*. USA: IEEE; 2022. p. 6437–6446.

[4] Ghosh P, Song J, Aksan E, Hilliges O. Learning human motion models for long-term predictions. In: *2017 International Conference on 3D Vision (3DV)*. USA: IEEE: 2017. p. 458–466.

[5] Jogendra NK, Maharshi G. BiHMP-GAN: Bidirectional 3D human motion prediction GAN. In: *Proceedings of the AAAI Conference on Artificial Intelligence*. USA: AAAI; 2019. p. 8553–8560.

[6] Cao Z, Gao H, Mangalam K, Cai Q-Z, Vo M, Malik J. Long-term human motion prediction with scene context. In: *European Conference on Computer Vision*. Germany: Springer; 2020. p. 387–404.

[7] Ma H, Li J, Hosseini R, Tomizuka M, Choi C. Multi-objective diverse human motion prediction with knowledge distillation. In: *Proceedings of the IEEE/CVF Conference on Computer Vision and Pattern Recognition*. USA: IEEE; 2022. p. 8161–8171.

[8] Yuan Y. Kitani K. Dlow: Diversifying latent flows for diverse human motion prediction. In: *European Conference on Computer Vision*. 2020.

[9] Mao W, Liu M, Salzmann M, Li H. Learning trajectory dependencies for human motion prediction. In: *International Conference of Computer Vision (International Conference of Computer Vision (ICCV))*. 2019.

[10] Li M, Chen S, Zhao Y, Zhang Y, Wang Y. Tian Q. Dynamic multiscale graph neural networks for 3D skeleton based human motion prediction. In: *Proceedings of the IEEE/CVF Conference on Computer Vision and Pattern Recognition*. USA: IEEE; 2020. p. 214–223.

[11] Cui Q, Sun H, Yang F. Learning dynamic relationships for 3D human motion prediction. In: *Proceedings of the IEEE/CVF Conference on Computer Vision and Pattern Recognition*. USA: IEEE; 2020. p. 6519–6527.

[12] Feng Y, You H, Zhang Z, Ji R, Gao Y. Hypergraph neural networks. In: *Proceedings of the AAAI Conference on Artificial Intelligence*. USA: AAAI; 2019. p. 3558–3565.

[13] Bai S, Zhang F, Torr PH. Hypergraph convolution and hypergraph attention. Pattern Recogn. 2021;110: Article 107637.

[14] Shi L, Zhang Y, Cheng J, Lu H. Skeleton-based action recognition with directed graph neural networks. In: *Proceedings of the IEEE/CVF Conference on Computer Vision and Pattern Recognition*. USA: IEEE; 2019. p. 7904–7913.

[15] Yadati N, Nimishakavi M, Yadav P, Nitin V, Louis A, Talukdar P. HyperGCN: A new method for training graph convolutional networks on hypergraphs. In: *NeurIPS*. USA: Curran Associates, Inc.; 2019. p. 644–656.

[16] Jiang J, Wei Y, Feng Y, Cao J, and Gao Y. Dynamic hypergraph neural networks. In: *International Joint Conferences on Artificial Intelligence*. USA: Morgan Kaufmann; 2019. p. 2635–2641.

[17] Tran L, Quan T, Mai A. PageRank algorithm for directed hypergraph. arXiv. 2019. https://doi.org/10.48550/arXiv.1909.01132

[18] Gui LY, Wang YX, Ramanan D, Moura JMF. Few-shot human motion prediction via meta-learning. In: *European Conference on Computer Vision*. Germany: Springer; 2018. p. 432–450.

[19] Anand G, Ankur M, Dan K, C. LG, Alexander O. A neural temporal model for human motion prediction. In: *Proceedings of the IEEE/CVF Conference on Computer Vision and Pattern Recognition*. USA: IEEE; 2019. p. 12116–12125.

[20] Cai Y, Huang L, Wang Y. Learning progressive joint propagation for human motion prediction. In: *European Conference on Computer Vision*. Germany: Springer; 2020.p. 226–242.

[21] Martinez J, Black MJ, Romero J. On human motion prediction using recurrent neural networks. In: *Proceedings of the IEEE/CVF Conference on Computer Vision and Pattern Recognition*. USA: IEEE; 2017. p. 2891–2900.

[22] Ashesh J, Amir RZ, Silvio S, Ashutosh S. Structural-RNN: Deep learning on spatio-temporal graphs. In: *Proceedings of the IEEE/CVF Conference on Computer Vision and Pattern Recognition*. USA: IEEE; 2016. p. 5308–5317.

[23] Guo X, Choi J. Human motion prediction via learning local structure representations and temporal dependencies. In: *Proceedings of the AAAI Conference on Artificial Intelligence*. USA: 2580-2587; 2019. p. 2580–2587.

[24] Liu Z, Wu S, Jin S, Liu Q, Lu S, Zimmermann R, Cheng L. Towards natural and accurate future motion prediction of humans and animals. In: *Proceedings of the IEEE/CVF Conference on Computer Vision and Pattern Recognition*. USA: IEEE; 2019. p. 10004–10012.

[25] Chiu HK, Adeli E,Wang B, Huang DA, Niebles JC. Action-agnostic human pose forecasting. In: *2019 IEEE Winter Conference on Applications of Computer Vision (WACV)*. USA: IEEE; 2019. p. 1423–1432.

[26] Mao W, Liu M, Salzmann M. Weakly-supervised action transition learning for stochastic human motion prediction. In: *Proceedings of the IEEE/CVF Conference on Computer Vision and Pattern Recognition*. USA: IEEE; 2022. p. 8151–8160.

[27] Maeda T, Ukita N. MotionAug: Augmentation with physical correction for human motion prediction. In: *Proceedings of the IEEE/CVF Conference on Computer Vision and Pattern Recognition*. USA: IEEE; 2022. p. 6427–6436.

[28] Guo W, Bie X, Alameda-Pineda X, Moreno-Noguer F. Multi-person extreme motion prediction. In: *Proceedings of the IEEE/CVF Conference on Computer Vision and Pattern Recognition*. USA: IEEE; 2022. p. 13053–13064.

[29] Gatta VL, Moscato V, Pennone M, Postiglione M, Sperlí G. Music recommendation via hypergraph embedding. In: *IEEE Transactions on Neural Networks and Learning Systems*. USA: IEEE; 2022. p. 7887–7899. 10.1109/TNNLS.2022.3146968 35143406

[30] Zhong C, Hu L, Zhang Z, Ye Y, Xia S. Spatio-temporal gating-adjacency GCN for human motion prediction. In: *Proceedings of the IEEE/CVF Conference on Computer Vision and Pattern Recognition*. USA: IEEE; 2022. p. 6447–6456.

[31] Diller C, Funkhouser T, Dai A. Forecasting characteristic 3D poses of human actions. In: *Proceedings of the IEEE/CVF Conference on Computer Vision and Pattern Recognition*. USA: IEEE; 2022. p. 15914–15923.

[32] Salzmann T, Pavone M, Ryll M. Motron: Multimodal probabilistic human motion forecasting. In: *Proceedings of the IEEE/CVF Conference on Computer Vision and Pattern Recognition*. USA: IEEE; 2022. p. 6457–6466.

[33] Zhang R, Zou Y, Ma J. Hyper-SAGNN: A self-attention based graph neural network for hypergraphs. In: *International Conference on Learning Representations (ICLR)*. USA: OpenReview; 2020.

[34] Diganta M. Mish: A self regularized non-monotonic neural activation function. In: *British Machine Vision Conference (BMVC)*. UK: British Computer Society; 2020. p. 1222–1236.

[35] Li B, Tian J, Zhang Z, Feng H, Li X. Multitask non-autoregressive model for human motion prediction. In: IEEE Transactions on Image Processing. USA: IEEE; 2020. p. 2562–2574. 10.1109/TIP.2020.3038362 33232232

[36] Diederik PK, Jimmy B. Adam: A method for stochastic optimization. In: *International Conference on Learning Representations (ICLR)*. 2015.

[37] Ionescu C, Papava D, Olaru V, Sminchisescu C. Human3.6M: Large scale datasets and predictive methods for 3D human sensing in natural environments. IEEE Transactions on Pattern Analysis and Machine Intelligence. 2014;36(7):1325–1339. 26353306 10.1109/TPAMI.2013.248

[38] Aliakbarian S, Saleh FS, Salzmann M, Petersson L, Gould S. A stochastic conditioning scheme for diverse human motion prediction. In: *Proceedings of the IEEE/CVF Conference on Computer Vision and Pattern Recognition*. 2020. p. 5223–5232.

[39] Li C, Zhang Z, Sun Lee W, Hee Lee G. Convolutional sequence to sequence model for human dynamics. In: *Proceedings of the IEEE/CVF Conference on Computer Vision and Pattern Recognition*. USA: IEEE; 2018. p. 5226–5234.

[40] CMU Graphics Lab: Carnegie-Mellon Motion Capture (Mocap) Database. 2003. http://mocap.cs.cmu.edu

[41] Gui LY, Wang YX, Liang X, Moura JMF. Adversarial geometry-aware human motion prediction. In: *European Conference on Computer Vision*. Germany: Springer; 2018. p. 786–803

[42] Timo vM, Henschel R, Black MJ, Rosenhahn B, Pons-Moll G. Recovering accurate 3D human pose in the wild using IMUs and a moving camera. In: *European Conference on Computer Vision*. Germany: Springer; 2018. p. 601–617.

[43] Dang L, Nie Y, Long C, Zhang Q, Li G. MSR-GCN: Multi-scale residual graph convolution networks for human motion prediction. In: *Proceedings of the IEEE/CVF International Conference on Computer Vision*. USA: IEEE; 2021. p. 11467–11476.

